# The Identification of Leidenfrost Phenomenon Formation on TiO_2_-Coated Surfaces and the Modelling of Heat Transfer Processes

**DOI:** 10.3390/ma17153687

**Published:** 2024-07-25

**Authors:** Monika Maziukienė, Nerijus Striūgas, Lina Vorotinskienė, Raminta Skvorčinskienė, Marius Urbonavičius

**Affiliations:** 1 Lithuanian Energy Institute, Laboratory of Combustion Processes, Breslaujos 3, LT-44403 Kaunas, Lithuania; monika.maziukiene@lei.lt (M.M.); lina.vorotinskiene@lei.lt (L.V.); raminta.skvorcinskiene@lei.lt (R.S.); 2 Lithuanian Energy Institute, Centre for Hydrogen Technologies, Breslaujos 3, LT-44403 Kaunas, Lithuania; marius.urbonavicius@lei.lt

**Keywords:** two-phase flow, vapour film, TiO_2_ coating, heat transfer, Leidenfrost phenomenon temperature

## Abstract

Experiments on specimen cooling dynamics and possible film boiling around a body are very important in various industrial applications, such as nucleate boiling, to decrease drag reduction or achieve better surface properties in coating technologies. The objective of this study was to investigate the interaction between the heat transfer processes and cooling dynamics of a sample in different boundary conditions. This article presents new experimental data on specimens coated with Al–TiO_2_ film and Leidenfrost phenomenon (LP) formation on the film’s surface. Furthermore, this manuscript presents numerical heat and mass transfer parameter results. The comparative analysis of new experiments on Al–TiO_2_ film specimens and other coatings such as polished aluminium, Al–MgO, Al–MgH_2_ and Al–TiH_2_ provides further detail on oxide and hydride materials. In the experimental cooling dynamics experiments, specimens were heated up to 450 °C, while the sub-cooling water temperatures were 14*‒20 °C (room temperature), 40 °C and 60 °C. The specimens’ cooling dynamics were calculated by applying Newton’s cooling law, and heat transfer was estimated by calculating the heat flux q transferred from the specimens’ surface and the Bi parameter. The metadata results from the performed experiments were used to numerically model the cooling dynamics curves for different material specimens. Approximated polynomial equations are proposed for the polished aluminium, Al–TiO_2_, Al–MgO, Al–MgH_2_ and Al–TiH_2_ materials. The provided comparative analysis makes it possible to see the differences between oxides and hydrides and to choose materials for practical application in the industrial sector. The presented results could also be used in software packages to model heat transfer processes.

## 1. Introduction

The development and modernization of electricity and heat generation technologies, with a focus on environmentally friendly technologies, are required to meet the goals of the Green Deal and achieve environmental stability. The main goal of these technologies is to produce energy without negatively impacting the environment [1]. In this regard, it is important to develop energy production technologies based on renewable resources [2].

The second issue that goes alongside new technology design is energy savings. Various bodies moving with kinetic energy encounter the problem of friction force, and within this, there is a need to reduce the drag coefficient (DK). Multiple methods have been applied to reduce the DK: streamlining or modifying the shape of the object [3]; applying overflow systems [4]; or covering the material’s surface with different coatings, which was found to reduce the drag coefficient by about 25% [5]. One of the methods used to decrease the drag coefficient is the application of bubble drag reduction (BDR), which can reduce drag by up to 80% [6] and allow 5% to 15% power savings [7]. When using a small bubble, the heat and mass transfer between the liquid and gas could be efficiently improved, resulting in a decreased drag coefficient [8]. This technology is known for advantages such as its safety, high efficiency and positive ecological impact [9]. One of the many alternative technologies for reducing the drag coefficient is the application of a vapour or gas layer to solid surfaces. Therefore, the Leidenfrost effect could be used as the basis for promising technology. A thin gas or vapour layer can change the slipping properties of a moving body. Investigations have shown that hydrodynamic drag can be reduced by up to 85% when comparing free-falling spheres at room temperature with spheres with a vapour layer formed around them [10].

The Leidenfrost phenomenon (LP) is vital for heat transfer processes. It only occurs when the body surface is hot enough and a vapour layer/film forms near the surface that does not permit water flow towards the surface [11]. The Leidenfrost temperature (LT) depends on many factors: surface properties [12,13], thermal properties [14,15], roughness [16,17], porosity [18] and topographic characteristics [19]. The literature provides many empirical correlations and methodologies for empirically evaluating the Leidenfrost temperature when considering the nature of the material [20] or the thermal properties of various substances [21]. Different studies have been performed to evaluate the effect of the surrounding liquid temperature on the minimal temperature that is necessary for the LP. Water is highlighted due to its lower threshold temperature (approximately 200 °C) [5]. Depending on the material, the LP did not occur in low surrounding water temperatures, and there is a lack of experiments on various materials [14]. Studies in the literature also suggest that stable vapour generation could be maintained on a free-falling sphere at high water temperatures, i.e., a T_w_ of ~85 °C to 95 °C [10,22]. Investigations have shown that the Leidenfrost temperature (T) is highly dependent on the material’s origin, surface structure, processing and oxidation. On processed zirconium alloy surfaces, pre-oxidation altered the critical heat flux formation and LPT by about 17% [23]. For other materials, it reached 320 °C when the surface was stainless steel [24], while it reached 188 to 428 °C for bare aluminium, depending on the conditions [19]. When the fluid is water, authors highlight the impact of the roughness of the aluminium material and the measured LT, which ranges from 158 to 185 °C [25].

One of the studied methods that could be applied to decrease the Leidenfrost temperature is surface structure modification. Some researchers have performed investigations showing that LPT depends on surface wettability and topography [26]. Attention should be given to the surface because the surface morphology and thickness are not uniform throughout a sample [27]. Surface porosity experiments have demonstrated that the LP is about 100 K and 200 K higher on 10% and 25% porosity alumina ceramic surfaces, respectively, compared with that on polished stainless steel [28,29]. To understand and evaluate various surfaces and the impact of their morphologies on LT formation, a variety of experiments must be performed with different materials to allow the processes occurring to be assessed.

This article presents new experimental results for specimens coated with Al–TiO_2_, and these results were compared with those of previous experiments on specimens coated with Al–MgH_2_ [30], Al–MgO [30], Al–TiH_2_ [31] and aluminium [31] films, which were performed in [30,31]. Empirically calculated and provided heat transfer parameters allow us to evaluate the differences between oxide and hydride coatings. The main novelty of this work is that it presents approximating polynomial equations using experimental result metadata. The approximating curves presented for each material allow the cooling dynamics trends for a particular substance to be visualized and the formation of the Leidenfrost phenomenon to be predicted.

## 2. Materials and Methods

### 2.1. Experimental Setup Section

**Figure 1:**
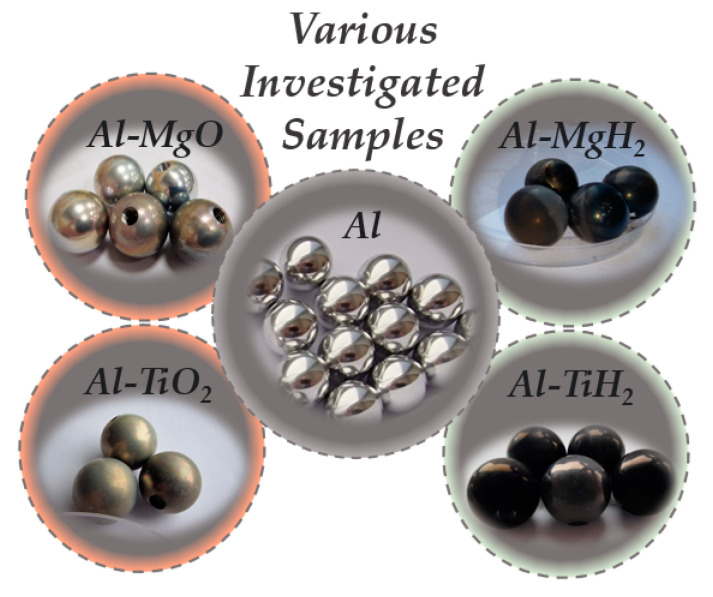
Experimental specimens.

The main goal of this study was to investigate the formation of the Leidenfrost effect on the TiO_2_-coated surface. The newly obtained experimental results for TiO_2_ were compared with those from previous experiments on polished aluminium [31], Al–MgO [30], Al–MgH_2_ [30] and Al–TiH_2_ [31]. The experiments in this work were performed with the main investigating material TiO_2_ and its spherical specimens (d = 19.05 mm) (Figure 1). Aluminium was chosen as the reference surface/coating. Experiments were performed to determine and evaluate the critical temperatures for Leidenfrost effect formation and its variation rate, which directly determines the evaporation process. Examples of the specimens used in the experiments are provided in Figure 1. The experiments were performed when investigating non-stationary heat transfer; the specimens were immersed in water at different temperatures: 14*–20 °C, 40 °C and 60 °C.

**Figure 2:**
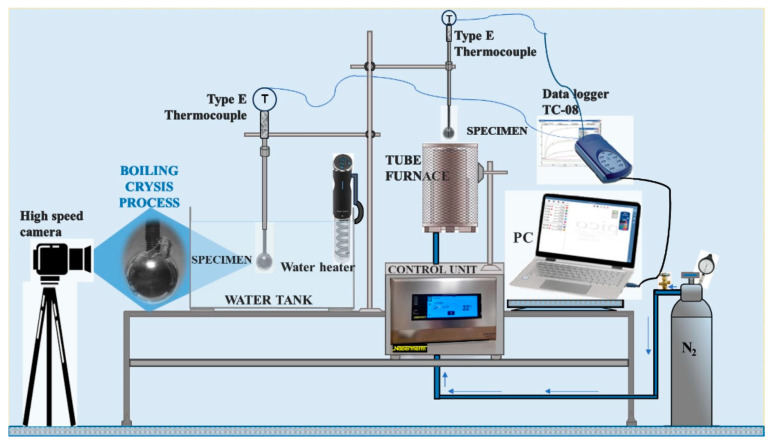
Experimental test setup for the determination of the critical Leidenfrost temperature.

The experiments were conducted in an experimental test section constructed in the laboratory. Its principal scheme is provided in Figure 2. The experimental test section was designed with four main parts: (1) a tube furnace, (2) a water tank, (3) a water heater and (4) a data recording system.

Experiments to determine the critical Leidenfrost temperature were performed in two consistent stages. Firstly, the spherical specimens were heated in the tube furnace (1) until they reached the desired temperature, which was 450 °C. In the second stage, the specimen cooling dynamics were observed in the water tank (3) and recorded with the data collection equipment (4). The variation in specimen surface temperature was captured using an E-type thermocouple (measuring in the range 50 ÷ 750 °C). One thermocouple was located inside the specimen before the opposite surface of the body at a distance of 1.5 mm. Copper paste was used to improve sealing and thermal conductivity. The temperature and the time taken to cool down to the desired temperature were monitored constantly using a “PICO TC-08 Thermocouple Data Logger”, which was connected to the thermocouple. The experimental data results were stored using “Data Logging software (PicoLog6)”. A 200 ms time interval was used for tracking the temperature variation. While performing the experiments, nitrogen gas was supplied to the tube furnace (2) at 2 L/min. When the specimens reached the desired temperature of 450 °C in the tube furnace (2), they were immersed in the water tank (3). The moment at which the specimen was fully submersed in the water was considered the zero time. The water temperature inside the tank (3) was maintained and changed using an electrical water heater with a temperature controller. The temperatures were set to 14*–20 °C, 40 °C and 60 °C.

To reduce errors, all the experiments were repeated three times under the same boundary conditions to obtain accurate results and to determine the deviation in the experimental results in a range of 2% to 11%. In the case of the Al–TiH_2_ and Al–TiO_2_ coatings, the maximum deviation from the mean did not exceed 12%. At the observed Leidenfrost point temperature, the deviation ranged between 2 and 9%. At lower temperatures, where intense bubble boiling occurs (not film boiling and not transition boiling regime), the deviation below a specimen temperature of 300 °C can be more varied (from 7 to 30%). However, the cooling dynamics below a specimen temperature of 300 °C were not considered significant in this part of the study, as bubble boiling occurs in a highly chaotic manner. For the Al–MgH_2_- and Al–MgO-coated specimens, the maximum deviation was lower than 10%. At the Leidenfrost temperature, the deviation was in the range of 2–4%. In the temperature range of 450 °C to 200 °C, the deviation varied from 2 to 10%. At lower temperatures, intense bubble boiling took place (not film boiling and not transition boiling regimes), and it was noticed that the deviation was more varied, from 4 to 17%, when the specimen temperature was 200 °C. However, deviations below a specimen temperature of 200 °C were not evaluated because this was not the focus of this research. For the aluminium specimens, the maximum deviation from the mean did not exceed 11%. The boundary conditions of the newly conducted experiments for Al–TiO_2_ materials are summarized and provided in Table 1. Previously obtained experimental data for polished aluminium [31], Al–MgO [30], Al–MgH_2_ [30] and Al–TiH_2_ [31] are detailed in [31] and [30]. These experiments’ metadata files were newly used for calculating and modelling the heat transfer processes, as well as when producing the generalized polynomial curves of Leidenfrost process formation for different materials.

### 2.2. Boiling Crisis and Leidenfrost Temperature Formation

The Leidenfrost point can be determined from the specimen cooling temperature graph. The cooling dynamics were recorded and the specimen’s surface cooling rate was calculated using the dt/dτ ratio. The latter expression of temperature velocity variation is used to identify the Leidenfrost temperature effect. It could be stated that if the temperature changes immediately after the specimen is immersed in water, vapour film does not form around it. On the other hand, if the temperature of the specimen remains high for a while, this could indicate that some film has formed that acts as an insulator from the surrounding environment.

### 2.3. Modelling Methodology of Heat Transfer

When the body temperature is higher than the surrounding temperature, energy losses can be described by radiation, conduction and convection processes. When comparing forced and natural convection, it is shown that radiation plays a significant role when forced convection is dominant. For such cases, various correlations could be applied. A significant impact of radiation could be observed when bodies are at temperatures higher than 600 °C. Therefore, the effect of radiation on cooling body dynamics is not considered in this work.

Theoretically, temperature changes between the body and its surroundings can be described using Newton’s law of cooling. The heat loss rate per unit area is proportional to the difference between the temperature of the body and its surrounding:

(1) $$\frac{1}{A}\frac{dQ}{dt}=h\left(T-T_{S}\right),$$

where Q is the heat flux, W; A is the area, m^2^; *t* is the time, s; *h* is the heat transfer coefficient, W/(m^2^ K); *T* is the temperature, °C; and *T_S_* is the temperature of the surroundings, °C.

As in Equation (1), the body cooling dynamics are derived from Newton’s law and are known as a time-dependent function. The temperature difference between the hot body and its surroundings decreases exponentially, and the cooling law can be written as follows [32]:

(2) $$T\left(t\right)=T_{S}+(T_{0}-T_{S})\cdot e^{-kt}$$

where  T(t) is the temperature of an object at a certain time, [°C]; *t* is the time, [s]; $T_{S}$ is the temperature of the surroundings, [°C]; and $T_{0}$ is the initial temperature of the heated object, [°C].

In cooling law, if the initial temperatures of a body, *T*_0_, and its surroundings, *T_S_*, are known, as well as the variation to a known temperature, *T_x_*, during the time, then Newton’s cooling law coefficient, *k*, can be expressed as follows [32]:

(3) $$k=\frac{1}{\tau }ln\left(\frac{T_{0-}T_{S}}{T_{x}-T_{S}}\right).$$

The provided equation is familiar and could be derived from the laws of thermodynamics and heat transfer. The main idea is to apply Newton’s law of cooling to samples that have different surface properties, such as polished aluminium, Al–TiO_2_, Al–MgO, Al–MgH_2_ and Al–TiH_2_. The cooling dynamics of other materials are very irregular. The obtained experimental data were used to calculate coefficient *k* empirically, to allow us to generalize the data and foresee the trend lines for different materials.

Practically, when considering the experimental data, the heat flow from the specimen surface could be expressed as follows [33]:

(4) $$Q=c_{k}\cdot m\cdot \sum \left(\Delta T/\Delta \tau \right)$$

where $c_{k}$ is the heat capacity of the sample, J/kgK; m is the mass of the specimen sample, kg; ΔT is the difference between two temperature readings of the data logs, K; and Δ*τ* is the time difference between two data logs, s.

If we assume that the heat flux balance condition is valid [33] and the amount of heat received by the specimen is equal to the heat transfer by specimen $Q_{1}=Q_{2}$, the heat flux transfer to the surroundings, W/m^2^, can be expressed as follows:

(5) $$q=\frac{c_{k}\cdot m\cdot \Delta T/\Delta \tau }{A}$$

When evaluating the heat transfer between a solid specimen and the overflown fluid, the Biot number is used, which is a fundamental value and shows the ratio between conduction and convection processes. When the specimen surface temperature is $T_{s,1}$ and is overflown by a fluid of temperature $T_{\infty }<T_{s,1}$, the intermediate temperature of the surface will be an intermediate value $T_{s,2}$:${\;T}_{\infty }<T_{s,2}<T_{s,1}$. Therefore, in terms of energy conservation, the surface energy balance could be written as follows [34]:

(6) $$\frac{\lambda A}{d}\left(T_{s,1}-T_{s,2}\right)=hA\left(T_{s,2}-T_{\infty }\right),$$

where λ is the thermal conductivity, W/mK; d is the characteristic length, m; A is the area, m^2^; and h is the heat transfer coefficient, W/m^2^K.

When expressing the ratio between internal conduction and external convection, Equation (6) is transformed to

(7) $$\frac{T_{S,1}-T_{S,2}}{T_{s,2}-T_{\infty }}=\frac{d/\lambda A}{1/hA}=\frac{R_{cond.}}{R_{convec.}}=\frac{h\cdot d}{\lambda }=Bi\;$$

## 3. Results and Discussion

### 3.1. Sample Cooling Dynamics and Vapour Film Formation

**Figure 3:**
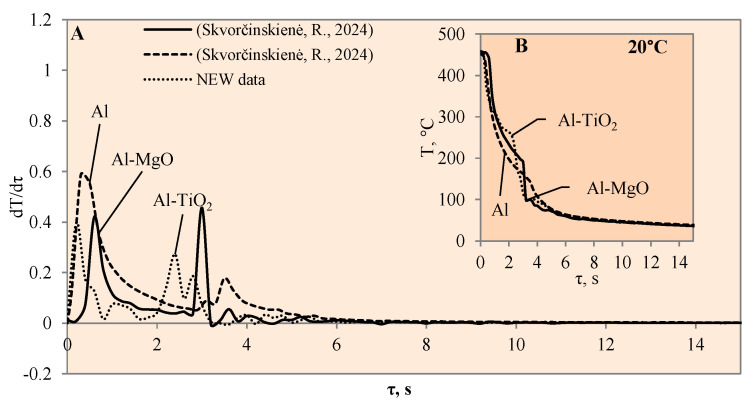
The cooling dynamics (**A**) and rate of cooling dynamics (**B**) for spherical samples of Al, Al–TiO_2_ and Al–MgO heated to 450 °C after immersion in water at 20 °C (the solid line corresponds to data obtained from reference [31], while the dashed line represents data from references [30,31]).

**Figure 4:**
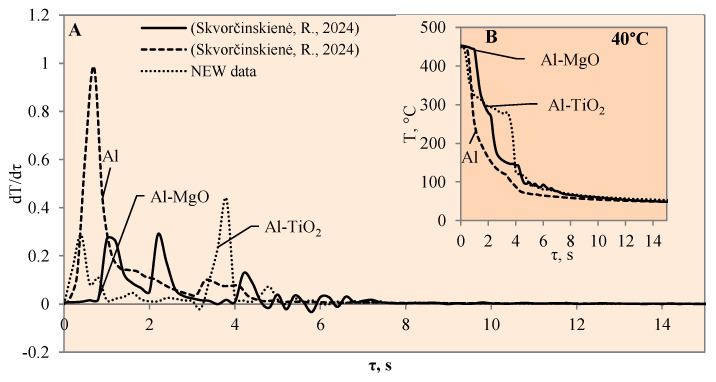
The cooling dynamics (**A**) and rate of cooling dynamics (**B**) for spherical samples of Al, Al–TiO_2_ and Al–MgO heated to 450 °C after immersion in water at 40 °C (the solid line corresponds to data obtained from reference [31], while the dashed line represents data from references references [30,31]).

**Figure 5:**
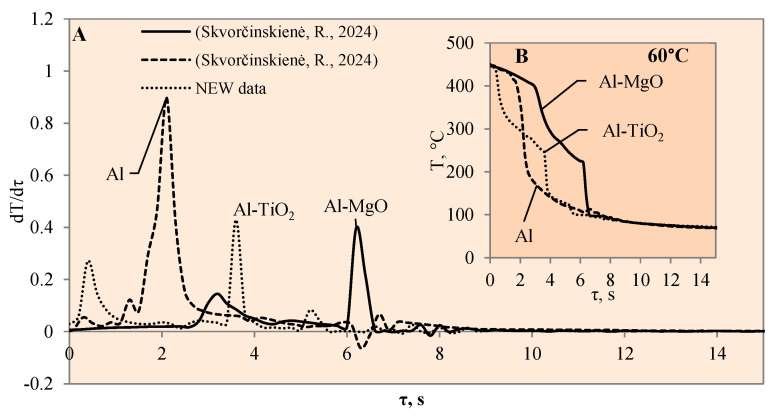
The cooling dynamics (**A**) and rate of cooling dynamics (**B**) for spherical samples of Al, Al–TiO_2_ and Al–MgO heated to 450 °C after immersion in water at 60 °C (the solid line corresponds to data obtained from reference [31], while the dashed line represents data from references references [30,31]).

**Figure 6:**
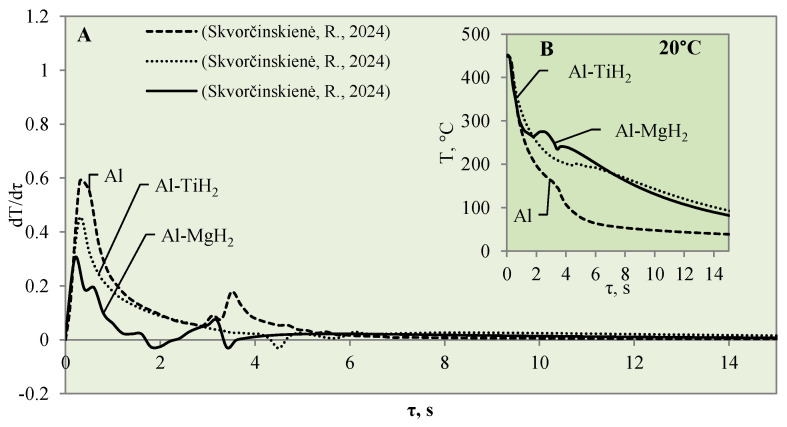
The cooling dynamics (**A**) and rate of cooling dynamics (**B**) for spherical samples of Al, Al–TiH_2_ and Al–MgH_2_ heated to 450 °C after immersion in water at 20 °C (the solid line corresponds to data obtained from reference [31], while the dashed line represents data from references [30,31], and the dotted line corresponds to reference [30]).

**Figure 7:**
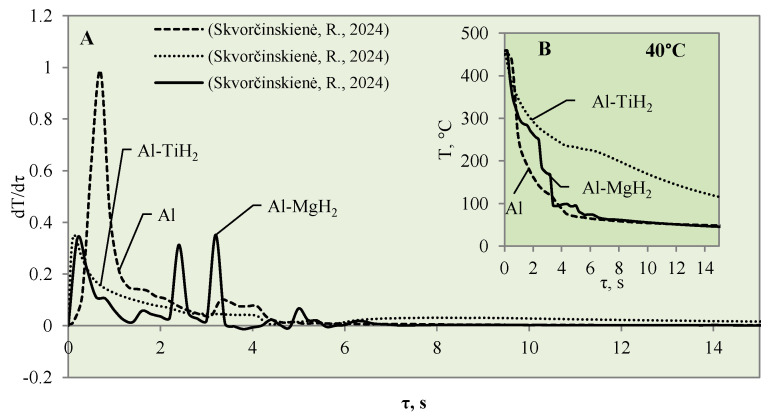
The cooling dynamics (**A**) and rate of cooling dynamics (**B**) for spherical samples of Al, Al–TiH_2_ and Al–MgH_2_ heated to 450 °C after immersion in water at 40 °C (the solid line corresponds to data obtained from reference [31], while the dashed line represents data from references [30,31], and the dotted line corresponds to reference [30]).

**Figure 8:**
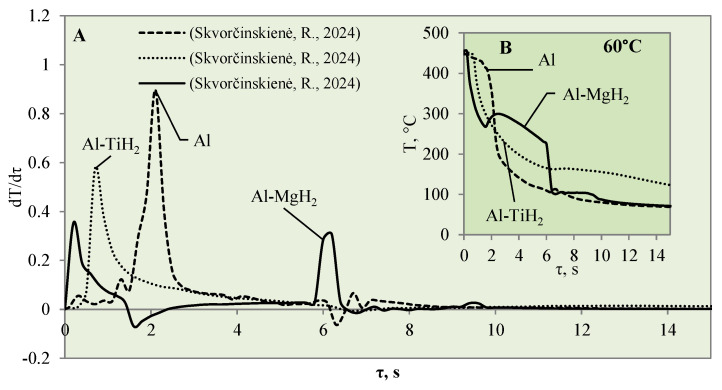
The cooling dynamics (**A**) and rate of cooling dynamics (**B**) for spherical samples of Al, Al–TiH_2_ and Al–MgH_2_ heated to 450 °C after immersion in water at 60 °C (the solid line corresponds to data obtained from reference [31], while the dashed line represents data from references [30,31], and the dotted line corresponds to reference [30]).

This study investigated the influence of hydrides and oxides on the occurrence of the Leidenfrost effect. In order to determine and evaluate the critical temperatures and the temperature variation rate, which determines the vapour generation process, experimental investigations were carried out with aluminium specimens coated with TiO_2_ (Al–TiO). The cooling dynamics of these specimens were compared with those of aluminium samples coated with MgO (Al–MgO), TiH_2_ (Al–TiH_2_), MgH_2_ (Al–MgH_2_) and polished aluminium films [30,31], which were investigated and described in our previous work. During the experiments, when unsteady heat transfer was investigated, the specimens were immersed in 20 °C, 40 °C and 60 °C water and the variation in their surface dynamics was recorded. The cooling rate calculated from the obtained experimental results is provided in Figure 3, Figure 4, Figure 5, Figure 6, Figure 7 and Figure 8 and allows us to more accurately identify the formation and lifetime of the vapour film forming around the specimen, as well as model the ongoing processes (i.e., describe them in theoretical equations). The analysis of the experimental results presented in this work and the comparison with those provided in our previous works [30,31] show that the cooling dynamics of the specimens coated with super-thin layers with different properties vary not only due to the temperature of the water but also due to the surface properties of the specimens (Figure 3, Figure 4, Figure 5, Figure 6, Figure 7 and Figure 8).

The experimental investigation performed with Al–MgO coatings [30] showed that, even if the water temperature is 20 °C, we can expect the Leidenfrost phenomenon to form but only when the right coating and surface temperature are selected (Figure 3). Therefore, it was decided to perform additional experiments with a TiO_2_ coating. Titanium dioxide was chosen due to its suitable chemical and heat transfer properties and breakdown potential.

When the Al–MgO specimen was heated to 450 °C and immersed in 20 °C water, a vapour film formed around the specimen. Although even the Leidenfrost effect was fixed in this experiment, the film that formed around the Al–MgO specimen was not stable and decomposed after the first millisecond. Since no surface temperature stabilization was observed on the sample, it is difficult to identify the formation of the Leidenfrost effect when analysing the resulting surface temperature variation curves (Figure 3B) [30]. From the calculated cooling rate of specimens for Al–MgO, we can accurately predict that the formed vapour film was thin because dT/dτ only reached 0.42 °C/s, while its lifetime was only about 0.2 s (Figure 3A). The experiments performed with specimens coated with Al–TiO_2_ films and heated up to 450 °C and immersed in 20 °C water did not produce the expected Leidenfrost effect and vapour film did not form. The cooling dynamics of all three samples provided in Figure 3B are very similar, which allows us to conclude that the vapour film formed around Al–TiO_2_ is extremely thin and this ultra-thin saturated vapour layer is not enough to form a barrier that inhibits the cooling of the specimen or the stabilization of the specimen surface temperature. When the polished aluminium specimens that were heated to 450 °C were immersed in 20 °C water, vapour film did not form. However, the cooling rate reached 0.6 °C/s (Figure 3B). It was observed that the Leidenfrost effect only forms on the polished aluminium specimen when the cooling rate is no less than >1 °C/s due to the high heat conductivity of aluminium, which reaches 237 W/m·K.

When the water temperature was increased up to 40 °C, the cooling dynamics of the specimens also became distinctive (Figure 4) and the Leidenfrost effect could be observed around both the Al–MgO and the polished aluminium specimens.

Although the vapour film around the polished aluminium sample collapsed after 0.1 s, it was thicker and stronger; therefore, after it collapsed, we obtained a fast temperature variation, which in this case was equal to 1 °C/s (dt/dτ =1 °C/s) (Figure 4A). The vapour layer that formed under the same conditions around the specimen covered with Al–MgO was equal to 0.3 °C/s. Although the vapour film that formed around the polished aluminium specimen was stronger, the Leidenfrost effect around the Al–MgO specimen persisted three times longer and lasted around 0.8 s. The specimens coated with Al–TiO_2_ and heated up to 450 °C did not form a vapour film after being immersed in the 40 °C and 60 °C water. However, the cooling dynamics of the Al–TiO_2_ specimens were different from those of polished aluminium Al and Al–MgO because the surface temperature variation slowed down in temperature ranges of 320 °C to 290 °C (in 40 °C water) and of 310 °C to 250 °C (in 60 °C water) (Figure 4B and Figure 5B).

The impact of hydride coatings for vapour film formation on the specimen’s surface cooling dynamics was described in previous publications [30,31]. However, this section provides a comparison between the two hydride coatings (Al–TiH_2_ and Al–MgH_2_) as well as with polished aluminium. The calculated and presented cooling rate curves for the specimen surfaces are the basis for the modelling of the boiling crisis process (Figure 6, Figure 7 and Figure 8).

After a detailed comparative analysis of the Al–TiH_2_, Al–MgH_2_ and polished aluminium specimens heated to 450 °C, it was noticed that when the specimens were immersed in 20 °C water during the first minute of the experiment, the surface cooling dynamics of all three samples were very similar and the initial temperature fell from 450 °C to 300 °C within 1.8 s (Figure 6B). After cooling down to 300 °C, the temperature cooling dynamics changed. The polished aluminium specimen further cools evenly in a parabolic manner, while the surface temperature of the specimen coated with hydrides stabilizes in the time range from the second to the sixth seconds; the temperature of Al–MgH_2_ increases slightly. A stagnation of or slight increase in surface temperature could be observed due to hydrogen formation and an intensive bubbling effect on the surface. The intensive bubbling around the hot specimen forms a thin vapour film, which complicates water flow toward the specimen and its cooling. When experimenting with the Al–MgH_2_ and Al–TiH_2_-coated samples, the formation of gas vapour bubbles was maintained throughout the process; therefore, these samples’ temperatures did not reach the temperature of the polished aluminium specimen even after 15 s. The Al–TiH_2_ and Al–MgH_2_ specimens’ surface temperatures were 50 °C higher. The experimental results obtained for the hydride-covered samples in 20 °C water were not as expected; no vapour film formed around the specimens, which can be clearly seen from the calculated cooling dynamics rate curves, where a sudden cooling of specimens starts from the first millisecond, i.e., immediately after contact with the water (Figure 6A).

The cooling dynamics for specimens covered with hydrides immersed in 40 °C water became even more distinctive from the first second in the investigation because a vapour film could be observed around the polished aluminium specimen (Figure 7A).

The vapour film that covered the polished aluminium remained stable for 0.1 s, while from the temperature change derivatives for Al–MgH_2_ and Al–TiH_2,_ we can notice that no stable vapour film formed around the specimens even during the first seconds. In this case (i.e., when water is 40 °C), the cooling dynamics for Al–TiH_2_ are very distinguished. The Al–TiH_2_ coated specimen heated to 450 °C and immersed in 40 °C water from the second second cooled down more slowly than the other two specimens; this was due to the hydrogen generation reaction on the surface of the TiH_2_ film, which starts at 450 °C. Because this reaction is endothermic, it could also impact temperature variation. In the experiments with the Al–MgH_2_ specimens, it was observed that the MgH_2_ film’s adhesion to the aluminium base was leaky and, in various experimental stages, the film peeled off and detached from the aluminium base. Due to this negative temperature impact of Al–MgH_2_ coatings, a very similar cooling dynamic was observed for the polished aluminium- and Al–MgH_2_ coated specimens, because from the sixth second of the experiment, the curves became completely uniform (no MgH_2_ film was left on the specimens). In the experimental investigations with Al–MgH_2_ and water heated to 60 °C, the temperature curves became identical from the sixth second of the experiment (Figure 8).

Raising the water temperature to 60 °C did not produce any effective changes in the experiments conducted with Al–MgH_2_ because the vapour film formed momentarily and lasted for only 1 ms after these specimens were immersed in water. It is difficult to analyse and assess such a Leidenfrost effect from the temperature stabilization curves; it was recorded optically in this case. As the MgH_2_ specimen cooled down and reached a surface temperature of 270 °C, an intensive bubbling effect started, which kept the surface temperature stable and even increased it by about 40 °C (Figure 8B). The bubbling effect, which lasted about 2–3 s, prevented water from reaching the specimen surface and did not allow it to cool, while the aluminium did not cool down over the entire cross section; therefore, a temperature gradient could be observed. When the bubbling effect slowed down, the specimen’s temperature also decreased, while from the sixth second of the investigation, a sudden change in specimen temperature could be observed (Figure 8B).

In contrast with the Al–MgH_2_ specimens, the Al–TiH_2_ specimens immersed in 60 °C water formed a vapour film that remained stable for 0.7‒1.6 s (Figure 8A). From the large number of experiments performed, it can be stated that the temperature change derivation curves do not only identify the formation of the Leidenfrost effect, but the size of the derivations could describe the stability and comparative thickness of the formed vapour film.

For example, the vapour film that formed around the polished aluminium was more stable and only collapsed after 1.6 s. The peak of the temperature derivatives for polished aluminium was equal to 1 °C/s, while the peak for Al–TiH_2_ reached only 0.6 C/s and the beginning of film decomposition started after 0.7 s.

A summary of the data on the formation of the Leidenfrost phenomenon on the specimen surface at different temperatures is provided in Table 2.

### 3.2. XRD Analysis Results

**Figure 9:**
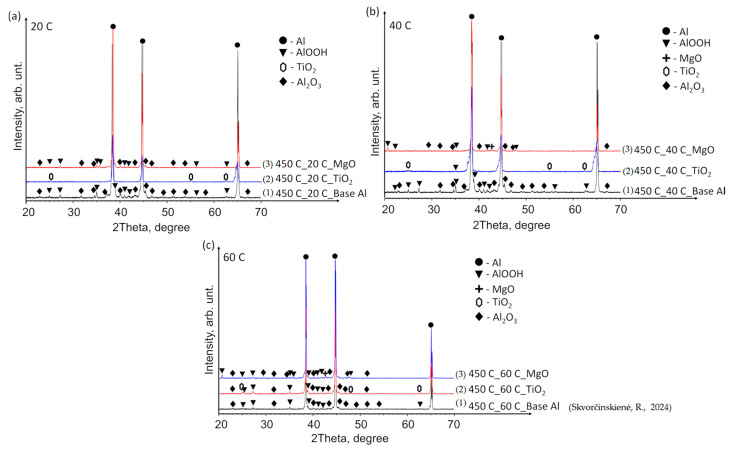
XRD patterns of (1) base Al, (2) TiO_2_ film deposited on an Al substrate and (3) MgO film deposited on an Al substrate preheated to 450 °C and subsequently immersed in water at temperatures of (**a**) 14*–20, (**b**) 40 and (**c**) 60 °C (the (1) line, in the case of 60 °C, is derived from reference [30]).

The X-ray diffraction (XRD) patterns of the base Al samples, preheated to 450 °C and immersed in 14*–20 °C, 40 °C and 60 °C water (Figure 9a–c), revealed minor peaks corresponding to aluminium oxyhydroxide (AlOOH, indicated by black triangles) and aluminium oxide (Al_2_O_3_, peaks marked with black diamonds). The elevated temperature facilitated the reaction of aluminium with moisture/water, forming AlOOH and Al_2_O_3_. However, the small size of the peaks indicates that the induced structural changes were not substantial.

Tiny TiO_2_ peaks were identified in all samples with a TiO_2_ film (indicated by hexagons). Notably, at a water temperature of 60 °C, peaks corresponding to AlOOH and Al_2_O_3_ (similar to those observed in the base Al samples) were detected in addition to TiO_2_ (Figure 9c). For comparison, the curve of the base Al sample preheated to 450 °C and immersed in water at a temperature of 60 °C was adopted from our previous paper [31]. The XRD pattern of the Al substrate with MgO film demonstrated a close similarity to that of the base Al. The as-deposited MgO film on the Al substrate exhibited an amorphous nature, while the MgO phase (indicated by crosses) was detected after the preheated samples were immersed in water at 40 and 60 °C (Figure 9b,c).

**Figure 10:**
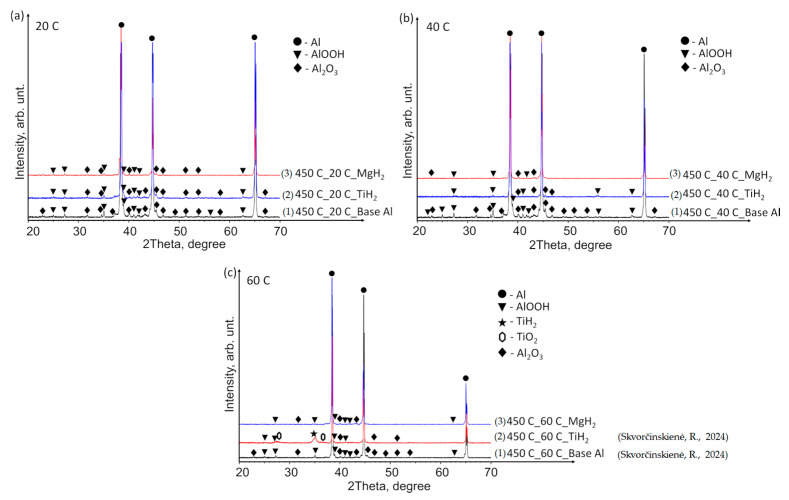
XRD patterns of (1) base Al, (2) TiH_2_ film deposited on an Al substrate and (3) MgH_2_ film deposited on an Al substrate preheated to 450 °C and subsequently immersed in water at temperatures of (**a**) 14*–20, (**b**) 40 and (**c**) 60 °C (the (1) and (2) lines, in the case of 60 °C, are derived from reference [30]).

Figure 10 presents the XRD patterns of the base Al, TiH_2_ films and MgH_2_ films deposited on aluminium substrates preheated to 450 °C and immersed in water at temperatures of 14*–20, 40 and 60 °C. The findings revealed the presence of the TiH_2_ phase (indicated by a black star peak) solely in the sample where the specimen with TiH_2_ film was preheated and immersed in water at 60 °C (Figure 10c). Small and broad peaks of TiO_2_ were also observed (Figure 10c, peaks indicated by hexagons). Neither TiH_2_ nor TiO_2_ was detected in the other specimens immersed in water at 14*–20 and 40 °C (Figure 10a,b). The aluminium specimens with deposited MgH_2_ film showed the same pattern as the base Al, with no observable trace of crystalline MgH_2_ or Mg-based phases in any of the cases.

### 3.3. Heat Transfer Modelling Results

The results for the formation of the Leidenfrost phenomenon and the appearance of film boiling for the specimens with different material coatings are presented in Table 2. It can be observed that the desired film boiling did not appear in the experiment performed with TiO_2_. Tong et al. also performed some experiments in nanotubes covered with TiO_2_ material and investigated the formation of the Leidenfrost phenomenon on the surface [35]; however, based on their observations, they claimed that the Leidenfrost temperature was higher than 480 °C or no Leidenfrost temperature could be achieved on such material. According to some studies, samples with TiO_2_ coatings can have a 68% better critical heat flux performance compared with uncoated surfaces [36]; however, the precise LT is not indicated in this study.

Heat fluxes from the specimen surfaces were calculated by applying the calculation methodology presented in Section 2 of this paper.

**Figure 11:**
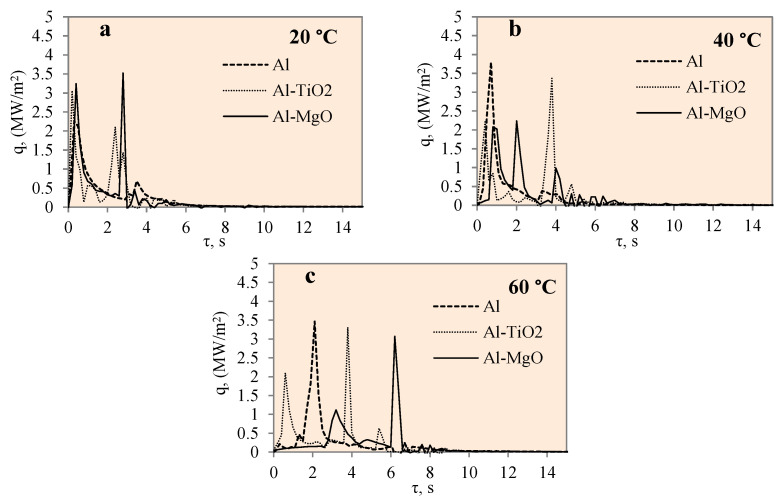
Heat flux variation on the surfaces of Al, Al–TiO_2_ and Al–MgO specimens. Heated specimen temperature 450 °C, water temperature (**a**) 20 °C, (**b**) 40 °C, (**c**) 60 °C.

Figure 11 represents the dependence of heat flux on time for the polished aluminium, Al–TiO_2_ and Al–MgO specimens during the cooling process at the different water temperatures of 14*–20, 40 and 60 °C. The heat flux change for different specimens was not uneven, as could be observed from a few high peaks.

The maximum heat flux values represent the end of a vapour film. The maximum heat flux peaks of the Al–MgO revealed and also confirmed the best vapour film formation results at different water temperatures. The heat flux values varied from ~0.5 to 3.6 MW/m^2^ during film boiling. The heat flux from the surface of Al–MgO reached very high values (almost 3.6 MW/m^2^) when the water temperature was equal to 20 °C. Experiments with polished aluminium revealed that vapour film had not formed when the water temperature was 14*–20 °C. When the water temperature was 40 or 60 °C, vapour film occurred and the heat fluxes varied from ~0.39 to 3.07 MW/m^2^·, respectively, during the film boiling period. While no vapour film formed around the Al–TiO_2_ specimens in any of the experiments conducted, the peaks of the heat flux curve show promising results at water temperatures of 40 and 60 °C. The highest flux values reached ~3.4 and ~ 3.3 MW/m^2^, respectively. The fact that no Leidenfrost phenomenon formed around Al–TiO_2_ could be explained by the conclusions of previous authors’ investigations [35,36], where higher specimen (above 450 °C) or surrounding water temperatures (above 60 °C) were needed.

**Figure 12:**
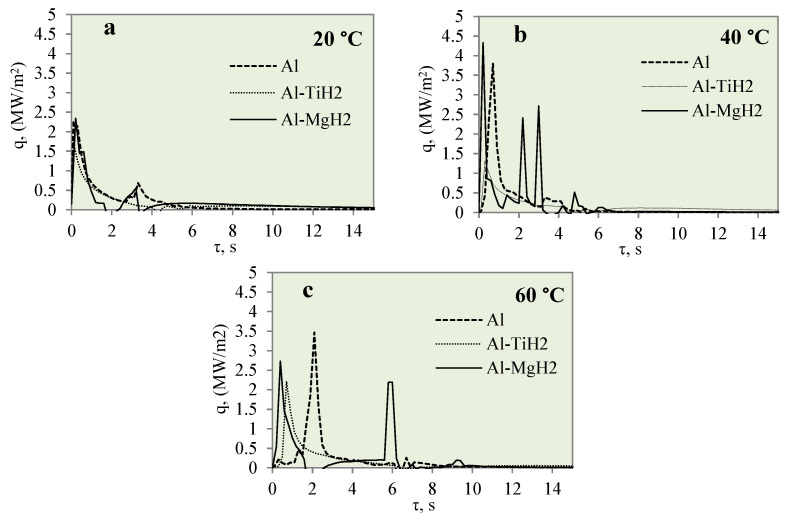
Heat flux variation for Al, Al–TiH_2_ and Al–MgH_2_ surfaces. Heated specimen temperature 450 °C, water temperature (**a**) 20 °C, (**b**) 40 °C, (**c**) 60 °C.

Figure 12 represents the dependence of heat flux on the specimen surface on time when investigating the cooling dynamics of the polished aluminium, Al–TiH_2_ and Al–MgH_2_ specimens. The cooling dynamics were investigated at different water temperatures of 14*–20, 40 and 60 °C. From Figure 12, it can be observed that the peaks show the lowest heat flux values when the water temperature is 14*–20 °C. No vapour film formed for any of the three different materials. The lack of vapour film formation could be influenced by a low heat transfer rate, which could be influenced by a low water temperature. When the water temperature was 40 °C, no Leidenfrost phenomenon was observed for the Al–TiH_2_ and Al–MgH_2_ specimens. However, the heat flux dynamics were quite the opposite. For the Al–MgH_2_-coated specimens, the highest heat flux values ranged from ~4.3 to ~2.7 MW/m^2^, respectively, in 40 and 60 °C water. For Al–TiH_2_, the maximum heat flux values reached only about ~0.8 and ~1.4 MW/m^2^ when the water temperatures were 40 and 60 °C, respectively. The low heat flux values for Al–TiH_2_ were affected by hydrogen separation and the intensive bubbling effect, which serves as an isolator around the hot specimen.

**Figure 13:**
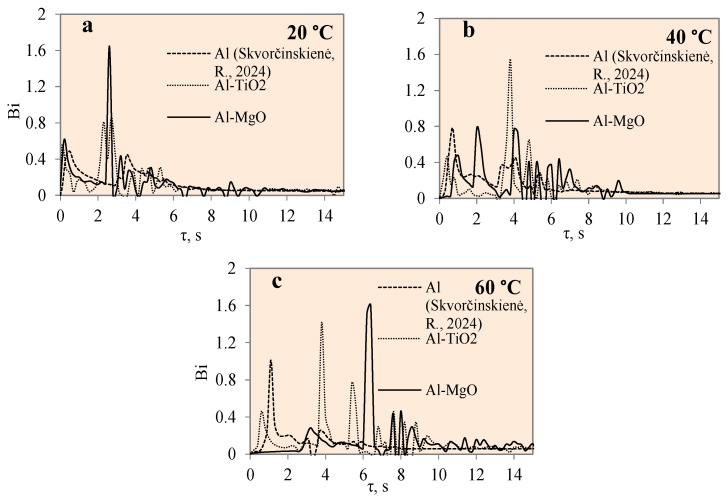
Biot number variation for Al, Al–TiO_2_ and Al–MgO specimens. Heated specimen temperature 450 °C, water temperature (**a**) 20 °C, (**b**) 40 °C, (**c**) 60 °C (the dashed line represents data from reference [30]).

Figure 13 presents the variation in Biot number over time for specimens coated with different materials, i.e., polished aluminium, Al–TiO_2_ and Al–MgO, and water temperatures of 14*–20, 40 and 60 °C, respectively. The Bi number is fundamental when evaluating conduction problems that involve surface convection effects. In this work, the Biot number for the specimens was calculated using Equation (7). The graphical interpretation of Bi number dynamics for the polished aluminium, Al–TiO_2_ and Al–MgO specimens is provided in Figure 13. When the condition Bi < 1 is satisfied, the resistance to conduction within the solid is much lower than the resistance to convection across the fluid boundary layer. The Al–TiO_2_ Bi was less than 1 and reached 0.9 when the water temperature was 14*–20 °C. At water temperatures of 40 °C and 60 °C, the Bi number for Al–TiO_2_ reached values of ~1.4 and ~1.5, respectively. This confirms that heat transfer by convection on the Al–TiO_2_ sample’s surface in warmer water is more intensive compared with heat transfer by conduction in a solid sample. The Bi number values for polished aluminium were investigated in [31]. For the Al–MgO samples, the Bi values were 1.6, 0.77 and 1.6 when the water temperatures were 14*–20, 40 and 60 °C, respectively. The results showed dominant heat transfer through convection and Leidenfrost formation in all cases. Regardless of the intensive heat transfer through convection, the Leidenfrost phenomenon was not observed on the Al–TiO_2_ samples and requires a broader investigation on boundary conditions. A summary of the results is presented in Table 3.

**Figure 14:**
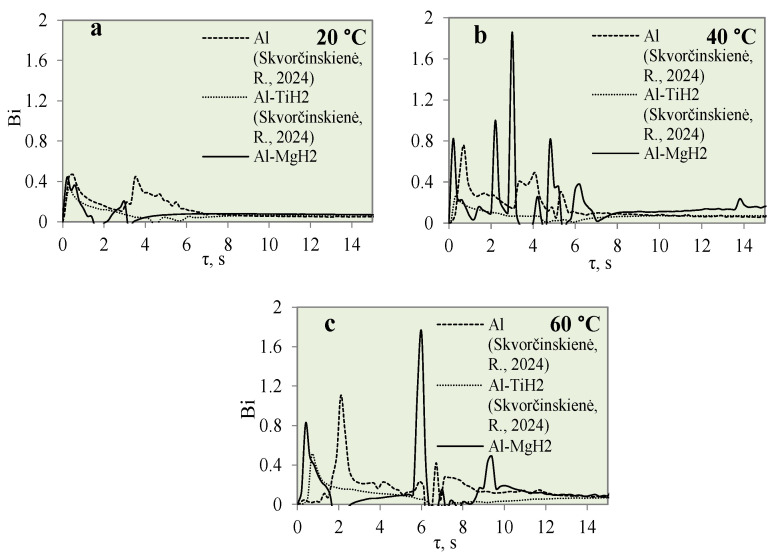
Biot number variation for Al, Al–TiH_2_ and Al–MgH_2_ specimens. Heated specimen temperature 450 °C, water temperature (**a**) 20 °C, (**b**) 40 °C, (**c**) 60 °C (the dashed and dotted lines represents data from reference [30]).

Figure 14 presents the graphical interpretation of the Bi number dynamics for the polished aluminium, Al–TiH_2_ and Al–MgH_2_ specimens.

Investigations of the Bi number for polished aluminium and Al–TiH_2_ were presented in [31]. The highest calculated Bi number values for Al–MgH_2_ specimens were 0.43, 1.8 and 1.74 when the water temperature was 14*–20, 40 and 60 °C, respectively. This shows that, in cooler water, heat transfer through conduction is more intensive than heat convection on the surface. Upon comparing the Bi values for hydrides and oxides, it could be observed that heat transfer through convection of the surface is greater for oxides than for hydrides. Therefore, it could be stated that hydride films of Al–TiH_2_ and Al–MgH_2_ have a better insulating property compared with oxides when evaluating the internal heat fluxes coming from the specimens through conduction.

**Figure 15:**
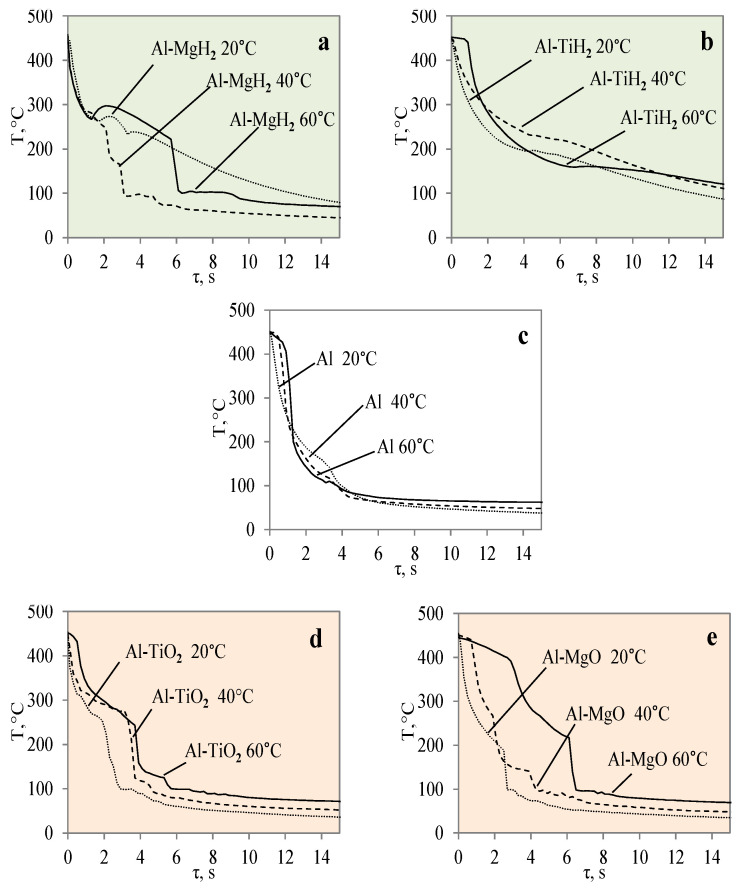
Approximations of surface temperature cooling dynamics for different materials: Al–MgH_2_ (**a**), Al–TiH_2_ (**b**), Al (**c**) Al–TiO_2_ (**d**), and Al–MgO (**e**) specimens when they were heated to 450 °C.

In this work, the obtained experimental data were used to derive and present empirical equations of Newton’s cooling law and to empirically calculate the cooling law coefficient *k* according to Equations (2) and (3) provided in the Section 2. The obtained experimental metadata results were used to generate cooling dynamics curves for the different material specimens. The numerically calculated cooling law equations are presented in Figure 15. The provided curves show the cooling dynamics for each investigated material, i.e., the Al, Al–TiO_2_, Al–MgO, Al–TiH_2_ and Al–MgH_2_ specimens, at water temperatures of 14*–20, 40 and 60 °C. The presented curves allow us to see trends not only in cooling but also in Leidenfrost temperature. From theory, it is known that variations in cooling surface temperature over time are highly dependent on the surface properties such as the surface material, roughness, porosity and thermal properties. This is also shown in Figure 15, where Al–TiO_2_ was highlighted for not forming the Leidenfrost phenomenon under the investigated conditions. Therefore, the curves in Figure 15d show a systematic dependence on temperature versus time.

Meanwhile, the curves for cases where the Leidenfrost phenomenon formed for aluminium, Al–MgO, Al–TiH_2_ and Al–MgH_2_ in Figure 15a–c,e show distinctive features that are highly dependent on the surface properties and the experimental boundary conditions. The approximated polynomial equations were derived for each material and are provided in Table 4. The obtained approximations could be used in future numerical and empirical models for modelling these materials’ cooling dynamics and in numerical calculations of the Leidenfrost phenomenon.

In the industrial sector, it is difficult to forecast the Leidenfrost temperature when working with different materials that are affected by high heat fluxes. Therefore, numerical calculations and evaluations must be performed to allow us to foresee the trends for various materials. This work presents approximated curves for each of the investigated materials: Al, Al–TiO_2_, Al–MgO, Al–TiH_2_ and Al–MgH_2_. The obtained approximations could be set in various modelling software packages and used in calculation methods to model the heat transfer processes of different materials. First, cooling dynamics experiments were performed with the specimens. In the second stage, the experiment’s metadata files were used to model the cooling dynamics and propose approximate cooling curves together with the Leidenfrost temperature. The provided equations make it possible to foresee the boundary conditions, i.e., body temperature or surrounding temperature, under which the Leidenfrost phenomenon forms. When modelling cooling processes, approximations make it possible to see the threshold of the phenomenon. The calculation results could also be applied practically to help in choosing materials more accurately.

## 4. Conclusions

This work presents new experimental results for the conditions for Leidenfrost phenomenon formation on an Al–TiO_2_ surface as well as a calculation and numerical simulation of the heat transfer parameter. A comparative analysis between hydrides and oxides was also performed using the experimental data obtained in this study and data on Al–MgH_2_, Al–MgO, Al–TiH_2_ and polished aluminium presented in [31] and [30]. All the specimens were heated to 450 °C, while the water temperature was 14*–20, 40 and 60 °C, respectively. The research and the comparative analysis focused on Al–TiO_2_. The following conclusions about Al–TiO_2_ were formed accordingly:

1. The experiments with the Al–TiO_2_ coating did not produce the expected Leidenfrost effect and no vapour film formed around the specimen at any of the water temperatures (14*–20, 40 or 60 °C). However, the cooling dynamics of the Al–TiO_2_ specimen showed a decrease in surface temperature variation in temperature ranges of 350 °C to 290 °C (in 40 °C water) and of 310 °C to 250 °C (in 60 °C water). It is likely that the heated specimen body temperature of 450 °C was too low for the appearance of the LP.
2. The performed XRD analysis showed that no substantial structural changes occurred in the polished aluminium specimens. The curves for the Al–TiO_2_, Al–MgO and Al–MgH_2_ films show high similarity to the curve for the base Al substrate. The results for Al–TiH_2_ show that aluminium was only present in the specimens that were heated up and immersed in water at 60 °C. The results could be affected by the adhesion process between the film and the base of the specimen.
3. The highest heat flux values on the surface of the Al–TiO_2_ specimens ranged from ~3.4 to ~3.3 MW/m^2^ without a Leidenfrost effect even being formed. The maximum heat flux values for the Al–MgO specimens were in the range of ~0.39 to 3.6 MW/m^2^. Al–MgO was also remarkable in terms of its Leidenfrost phenomenon formation at all water temperatures. The maximum heat flux peak values for Al–TiH_2_ were in the range of ~0.8 to ~1.4 MW/m^2^, while for Al–MgH_2_, the highest heat flux values ranged from ~4.3 to ~2.7 MW/m^2^, respectively.
4. The calculated Bi number values for TiO_2_ increased from 0.9 to 1.4 to 1.5 at water temperatures of 20, 40 and 60 °C, respectively. Increasing the water temperature impacts heat transfer and results in the dominance of convection transfer on the surface of the specimen over heat transfer via internal conduction. The higher Bi values of the Al–TiO_2_ and Al–MgO oxide materials show that the hydrides Al–TiH_2_ and Al–MgH_2_ have superior insulating properties.
5. Approximated polynomial equations were newly proposed for each of the investigated materials, Al, Al–TiO_2_, Al–MgO, Al–TiH_2_ and Al–MgH_2_, and their cooling dynamics. The presented equations make it possible to foresee the cooling rate for a given material, predict the formation of the Leidenfrost temperature and indicate the threshold of the phenomenon. The obtained results could be used for heat and mass transfer models and for selecting the right materials in the industrial sector.

## Figures and Tables

**Table 1:** Boundary conditions of conducted experiments.

Surface of Specimens	Specimen Temperature, °C	Water Temperature, °C	Measurement Time, s
Al–TiO_2_	450	20	15
		40	
		60	

**Table 2:** Leidenfrost temperature (critical temperature).

Materials	Specimens, Temperature, °C	Water Temperature, °C		
		14*–20	40	60
polished Al	450	No ^v^	374 ^v^	331 ^v^
Al–TiO_2_		No	No	No
Al–MgO		439 ^v^	441 ^v^	373 ^v^
Al–TiH_2_		No ^v^	No ^v^	385 ^v^
Al–MgH_2_		No ^v^	No ^v^	384 ^v^

^v^—indicates that the experimental results were presented in [30,31].

**Table 3:** A summary of Biot number variations.

T_water_,° C	T_speciment_,°C	Bi_Al_	Bi_Al–TiO_2__	Bi_Al–MgO_	Bi_Al–TiH_2__	Bi_Al–MgH_2__
14*–20	450	0.46	0.9	1.6	0.33	0.43
40		0.93	1.4	0.77	0.25	1.8
60		1.05	1.5	1.66	0.50	1.74

**Table 4:** Approximation lines for specimens’ cooling dynamics.

Material	Approximation
Al	T_s_ = 5 × 10^−25^τ^6^ − 1·10^−19^τ^5^ + 9 × 10^−15^τ^4^ − 4 × 10^−10^τ^3^ + 9 × 10^−6^τ^2^ − 0.0844τ + 345.48
Al–TiO_2_	T_s_ = 10^−24^ × τ^6^‒ 2·10^−19^τ^5^ + 2 × 10^−14^τ^4^ ‒ 6 × 10^−10^ τ^3^+ 1 × 10^−5^τ^2^ ‒ 0.1063τ + 461.57
Al–MgO	T_s_ = 3 × 10^−25^τ^6^ − 8·10^−20^τ^5^ + 7 × 10^−15^τ^4^ − 3 × 10^−10^τ^3^ + 8 × 10^−6^τ^2^ − 0.0861τ + 408.54
Al–TiH_2_	T_s_ = 2 × 10^−24^τ^6^ − 4·10^−19^τ^5^ + 2 × 10^−14^τ^4^ − 8 × 10^−10^τ^3^ + 1 × 10^−6^τ^2^ − 0.1006τ + 462.09
Al–MgH_2_	T_s_ = 6 × 10^−26^τ^6^ − 2·10^−20^τ^5^ + 2 × 10^−15^τ^4^ − 1 × 10^−10^τ^3^ + 4 × 10^−6^τ^2^ − 0.0553τ + 388.53

## Data Availability

The data presented in this study are available upon request from the corresponding author. The data are not publicly available due to privacy.
